# Testing Interactions in Multinomial Processing Tree Models

**DOI:** 10.3389/fpsyg.2019.02364

**Published:** 2019-11-01

**Authors:** Beatrice G. Kuhlmann, Edgar Erdfelder, Morten Moshagen

**Affiliations:** ^1^Department of Psychology, School of Social Sciences, University of Mannheim, Mannheim, Germany; ^2^Department of Psychology, Ulm University, Ulm, Germany

**Keywords:** multinomial processing tree models, interactions, parametric order constraints, associative deficit hypothesis, cognitive aging

## Abstract

Multinomial processing tree (MPT) models allow testing hypotheses on latent psychological processes that underlie human behavior. However, past applications of this model class have mainly been restricted to the analysis of main effects. In this paper, we adopt the interaction concept as defined in log-linear models and show why it is appropriate for MPT models. We then explain how to implement and test ordinal and disordinal two-way interaction hypotheses in MPT models. We also show how our method generalizes to higher-order interactions involving three or more factors. An empirical example from source memory and aging demonstrates the applicability of this method and allows for directly testing the associative deficit theory that age differences are larger in associative (e.g., source) memory as opposed to item memory. Throughout the paper, we explain how most analytic steps can be easily implemented in the freely available software multiTree.

## Introduction

Psychologists are typically interested in internal processes (e.g., cognitions and emotions) that drive behavior but are not directly observable. Multinomial processing tree (MPT) models are stochastic models that, based on observable participant responses, allow for estimation of the probabilities of such unobservable processes taking place or not. Developed in the 1980s and 1990s (Batchelder and Riefer, 1980, 1986, 1999; Riefer and Batchelder, 1988; Hu and Batchelder, 1994), they are currently widely used in several branches of psychological research (see Erdfelder et al., 2009, for a comprehensive review). However, so far almost all applications of MPT models in psychology involve simple parameter comparisons across experimental conditions or groups of participants, thus testing only main effects on model parameters, arguably because standard MPT parameter tests do not readily allow to test interactions. In the present paper, we explain how MPT models can be reparameterized to allow for testing interaction hypotheses. We provide an easy-to-follow introduction and an application example from cognitive aging on how to implement parameter constraints to test two-way (and higher-order) interaction hypotheses, using the software multiTree (Moshagen, 2010).

### A Brief Introduction to MPT Models

MPT models are used to analyze categorical data such as response frequencies. In contrast to general-purpose data analysis techniques for categorical data (e.g., log-linear models, Read and Cressie, 1988; Agresti, 2002), MPT models are tailored to a specific psychological research paradigm and are based on substantive theoretical work regarding the processes involved in the psychological phenomenon investigated in this paradigm (e.g., Bayen et al., 1996; Rummel et al., 2011). Thereby, MPT models can be used to evaluate theories of a given psychological phenomenon as well as to estimate probabilities of the latent processes specified in the theory. In this section, we provide a brief introduction to MPT models and standard inferential tests available for MPT model parameters (for technical details, see Riefer and Batchelder, 1988; Hu and Batchelder, 1994; Batchelder and Riefer, 1999).

**Figure 1:**
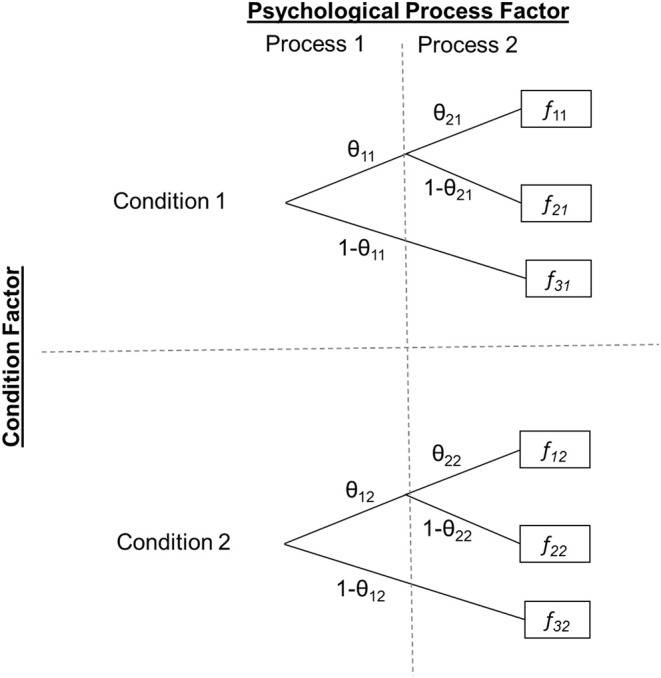
Generic two-by-two experimental design with MPT parameters as dependent variables. Two MPT parameters (θ_1·_, θ_2·_) representing the probabilities of two distinct psychological processes are estimated within two (within- or between-subjects) conditions, resulting in a total of four parameters. The first subscript indicates the process parameter (1, 2) and the second the condition level (1, 2). *f*_*ic*_ denotes response *i, i* = 1, …, 3, observed in condition *c* (1 or 2).

The upper half of Figure 1 displays a very simple generic MPT model containing two parameters, θ_1_ and θ_2_. For now we will ignore the second subscript, which indicates that the model and its two parameters are estimated separately for two groups (e.g., experimental conditions). The boxes to the right indicate observable categorical participant responses in a psychological paradigm, for example “Remember,” “Know,” and “New” responses in a Remember-Know recognition task (Gardiner, 1988), with three distinct responses in total (*f*_1_, *f*_2_, and *f*_3_ again observed separately per condition). The core idea of any MPT model is to relate the probabilities of such observable responses to the probabilities of unobservable psychological processes (measured by parameters θ_1_ and θ_2_). Specifically, in this simplified generic model^1^, the three observable response probabilities relate to the model parameters as follows:

(1) $$\begin{array}{l} \text{p}\left({\text{f}}_{1}\right)=\theta_{1}\cdot \theta_{2}\\ \text{p}\left({\text{f}}_{2}\right)=\theta_{1}\cdot \left(1-\theta_{2}\right)\\ \text{p}\left({\text{f}}_{3}\right)=\left(1-\theta_{1}\right) \end{array}$$

To estimate these parameters, MPT models assume that the frequencies of the observable responses follow a (product) multinomial distribution. This distribution is then described in terms of the model parameters by replacing the multinomial response probabilities with the right side of the corresponding model equation. Thus, any parameter in the model is an unknown probability varying in the real interval [0, 1]. Parameter estimates are typically obtained by means of maximum likelihood estimation, more specifically, by minimizing the log-likelihood ratio goodness-of-fit statistic *G*^2^(*df* ) using the expectation-maximization algorithm (Hu and Batchelder, 1994). Given that the model holds and certain regularity conditions are met (Read and Cressie, 1988, Chapter 4), the resulting minimum *G*^2^(*df* ) is asymptotically χ^2^(*df* ) distributed, with the degrees of freedom (*df* ) corresponding to the number of independent response probabilities minus the number of estimated parameters. The obtained model fit statistic can thus be compared to a χ^2^(*df* ) distribution to evaluate the null hypothesis (H_0_) that the model fits the data. The H_0_ of model fit is rejected when the observed *G*^2^(*df* ) fit statistic falls in the upper α·100% of the corresponding χ^2^(*df* ) reference distribution (typically, α = 0.05 or α = 0.01) and retained otherwise. Several computer programs are freely available for model fitting and parameter estimation (Hu and Phillips, 1999; Stahl and Klauer, 2007; Moshagen, 2010; Singmann and Kellen, 2013; Heck et al., 2018).

To test more specific hypotheses concerning the model parameters, restrictions can be imposed reflecting the H_0_ that a model parameter is either equal to (1) a constant value (e.g., chance level for guessing between two options = 0.50) or (2) another parameter in the model. It is then tested whether this restriction significantly worsens model fit by comparing the fit of the restricted model [$G_{\text{r}}^{2}$(*df*_r_)] to the fit of the unrestricted model [$G_{\text{u}}^{2}$(*df*_u_)], based on a χ^2^ difference test [i.e., Δ*G*^2^(*df*_r_-*df*_u_) = $G_{\text{r}}^{2}$(*df*_r_) – $G_{\text{u}}^{2}$(*df*_u_)]. If model fit is significantly worsened by the restriction, the H_0_ that the parameter restriction holds is rejected.

However, neither of these standard MPT parameter tests allows for testing interactions in multifactorial designs. To illustrate this problem, consider the two-factorial experimental design exemplified in Figure 1. First, there is a Condition factor with two levels (Condition 1, Condition 2). Our generic MPT model is assumed to hold in each of these two conditions with presumably different parameter values. Second, there is a Process factor representing the psychological mechanism of interest, again with two levels (θ_1_, θ_2_). A standard MPT parameter test can be used to test for a main effect of the Condition factor on the psychological processes by restricting θ_1_ and θ_2_ to be equal across conditions (θ_11_ = θ_12_ and θ_21_ = θ_22_). Alternatively, one can test for a main effect of the psychological process by simultaneously restricting θ_1_ and θ_2_ to be equal within each condition (θ_11_ = θ_21_ and θ_12_ = θ_22_). Additionally, the simple main effects of the Process factor can be assessed for each condition separately or, if more appropriate, the simple main effects of the Condition factor separately for each psychological process. However, all these tests do not straightforwardly implement the test of an interaction of the Condition factor with psychological process. For example, a theory might predict that there should be a general condition main effect with Condition 1 outperforming Condition 2 but that this effect is moderated by the type of psychological process, such that the condition effect is more strongly pronounced in the process represented by parameter θ_2_ than in the process represented by θ_1_. In this case, one could test the effect of condition on both parameters but would lack a test for comparing the strengths of this condition effect between the two processes.

## Method

In the following, we will derive a method to test interactions on MPT model parameters. We will primarily focus on two-way interactions but later explain how our method generalizes to testing higher-order interactions. For now, we will assume the experimental design with two factors illustrated in Figure 1 comprising a Condition factor with two levels (Condition 1, Condition 2; within- or between-subjects) and a Process factor with two levels (Parameter θ_1·_, Parameter θ_2·_). Note that, in principle, the second factor could also reflect any different grouping (such as an experimentally manipulated within or between condition factor). In this case, only one MPT model parameter would be of interest that is estimated in the four cells resulting from fully crossing both condition factors.

### Appropriate Interaction Concept for MPT Models

When assessing interactions, a distinction needs to be made between interactions as defined in analysis of variance (ANOVA) models and interactions as defined in Log-Linear Models (LLM). In our Condition × Process design, a null interaction in the ANOVA sense would refer to invariance of parameter *differences* between the levels of one factor across the levels of the second factor. That is, a null interaction would mean that the difference, δ, between conditions is identical on both parameters. Likewise, the difference between the two process parameters would need to be the same across conditions. In the following, we will demonstrate that due to the nature of MPT model parameters, which represent probabilities and are thus bounded by 0 and 1, this linear interaction concept is not suited for this type of models and may falsely imply the presence of an interaction in the presence of strong main effects. To demonstrate this, let us assume, without loss of generality, that factor levels are arranged such that moving from Level 1 to Level 2 on either factor results in a constant decrease of the corresponding parameters under the null interaction model. Therefore, the following would need to hold for a null interaction of condition and process in the ANOVA sense:

(2) $$\begin{array}{l} \theta_{11}-\theta_{12}=\theta_{21}-\theta_{22}=\delta_{\text{C}} \end{array}$$

(3) $$\begin{array}{l} \theta_{11}-\theta_{21}=\theta_{12}-\theta_{22}=\delta_{\text{P}} \end{array}$$

with δ_C_ and δ_P_ indicating the main effects of the two factors [Condition (C), Process (P)], 0 ≤ δ_C_ ≤ 1 and 0 ≤ δ_P_ ≤ 1. Therefore, under the null hypothesis of no interaction, parameter θ_22_ would be affected by both main effects as follows

(4) $$\begin{array}{l} \theta_{22}=\theta_{11}-\delta_{\text{C}}-\delta_{\text{P}}. \end{array}$$

Because θ_22_, like all MPT parameters, represents a probability, it must lie within [0, 1]. Consequently, the following restriction would be imposed on the main effects of condition and process for a null interaction to be present:

(5) $$\begin{array}{l} \left(\delta_{\text{C}}+\delta_{\text{P}}\right)\leq \theta_{11} \end{array}$$

A weird consequence of (5) is that strong main effects would automatically imply a violation of (5) and thus an interaction in the ANOVA sense. If, for example, θ_11_ = 0.70 and θ_12_ = 0.10 (i.e., δ_C_ = 0.60), an interaction of condition and process would automatically be implied whenever the Process factor has a main effect larger than δ_P_ = 0.10 to ensure that θ_22_ ≥ 0. More generally, if the largest parameter is quite small (e.g., 0.05) not much shrinkage is possible if defined as a difference (i.e., 0 ≤ δ ≤ 0.05). In the standard ANOVA framework, in contrast, main effects and interactions could vary independently because parameters are not bounded by 0 and 1.

This obvious problem can be avoided in MPT modeling by adopting the LLM interaction concept in which a null interaction refers to invariance of parameter *ratios* across the levels of the second factor. That is, in terms of the 2 × 2 model with a Condition and a Process factor, a null interaction in the LLM sense would imply:

(6) $$\begin{array}{l} \theta_{12}/\theta_{11}=\theta_{22}/\theta_{21}=\alpha_{\text{C}} \end{array}$$

(7) $$\begin{array}{l} \theta_{21}/\theta_{11}=\theta_{22}/\theta_{12}=\alpha_{\text{P}} \end{array}$$

In this case, assuming no LLM-type interaction, θ_22_ would be:

(8) $$\begin{array}{l} \theta_{22}=\theta_{11}\cdot \alpha_{\text{C}}\cdot \alpha_{\text{P}} \end{array}$$

Crucially note that, in order to keep all MPT parameter values within [0, 1], main effects must be represented as ratios α_C_ and α_P_ with the larger parameter values always appearing in the denominator. Most importantly, however, an interaction effect is not automatically implied when α_C_ and α_P_ take on extreme values in [0, 1], because main effects are multiplicative rather than additive under the null interaction hypothesis in the LLM sense. Thereby, even strong main effects can occur (i.e., α parameters close to 0) without implying an interaction. For the example given earlier, if θ_11_ = 0.70 and θ_12_ = 0.10 (i.e., α_C_ = 0.14), the difference θ_21_ – θ_11_ could still exceed 0.10 without necessarily implying an interaction. For example, if θ_12_ = 0.40 (i.e., α_P_ = 0.57), a null interaction in the LLM sense would require θ_22_ = 0.06, a valid parameter value. Thus, main effects and interactions would not be artificially confounded. Likewise, the α parameters are not restricted by the value of the original parameter; even if the largest parameter is quite small, the α parameter can, in principle, be of any value between 0 and 1. Hence, we will adopt the LLM interaction concept, because it is better tailored to multinomial models. For brevity, we refer to this latter concept of “interaction as defined in LLM” simply as “interaction” in what follows^2^.

### Estimating MPT Parameter Ratios via Parametric Order Constraints

Testing an interaction hypothesis essentially entails comparing a factor's simple main effect under different conditions. As reasoned above, it is crucial that the main effects are expressed as *parameter ratios* (rather than differences) to test an interaction in the LLM sense. In order to perform this type of comparison with the parameter tests available in MPT models, it is thus necessary to introduce parameters that quantify a factor's simple main effect in each possible condition as a ratio. Then, these ratio parameters can be compared across conditions to assess whether an interaction is present (i.e., the factor's simple main effect differs depending on conditions) or not.

Knapp and Batchelder (2004) introduced reparameterizations of MPT models that allow estimating parameter ratios via *parametric order constraints*. As described earlier, all estimated MPT model parameters vary in [0, 1] when freely estimated but can be restricted to be equal to a constant or to another parameter. In contrast, parametric order constraints allow to implement restrictions that still let the constrained parameter vary freely but only within a restricted range of values. For example, the restriction 0 ≤ θ_1_ ≤ θ_2_ ≤ 1 constrains the parameter θ_1_ to vary only in [0, θ_2_] instead of [0, 1], with the upper boundary being determined by the value of another model parameter, θ_2_. Once a parametric order constraint, such as θ_1_ ≤ θ_2_, has been defined and a MPT model has been reparameterized to reflect this constraint, a new parameter α is estimated that reflects the shrinkage of θ_1_ relative to θ_2_. Crucially, we propose that this shrinkage parameter α can be used to test interactions because it reflects the *relative* change in parameters and thus, in essence, represents the ratio α = θ_1_/θ_2_.

Knapp and Batchelder (2004) introduced two equivalent reparameterization methods for MPT models to assess parametric order constraints. We will focus on their Method A, which has been implemented in the software multiTree (Moshagen, 2010). This method replaces a parameter θ by a novel parameter α such that α reflects *decreases* in θ, allowing the implementation of a constraint that restricts one parameter (e.g., θ_1_) to be *smaller than or equal to* a second parameter (e.g., θ_2_). Note that such a reparameterization does not change the dimensionality of the model (i.e., the number of parameters) and thus does not change the *df* of the model test. Also, note that this reparameterization is data-equivalent to the original model whenever the implemented order constraint actually holds in the observed data.

To apply this method for purposes of testing interactions, data from at least two (within- or between-subjects) conditions are required across which a parameter may change. Returning to our two-factorial example illustrated in Figure 1, there is a Condition factor with two levels/groups (e.g., younger and older adults). As before, let us assume that Condition 1 outperforms Condition 2 on both psychological processes measured by the model's parameters θ_1·_and θ_2·_. Via Knapp and Batchelder's method A, we can replace θ_12_ and θ_22_ by two new parameters, α_C, θ1_ and α_C, θ2_, representing the shrinkage factors for each process in Condition 2 compared to Condition 1. In the reparameterized model, the parameters of Condition 2 are thus reparameterized as follows:

(9) $$\begin{array}{l} \theta_{12}=\theta_{11}\cdot \alpha_{\text{C}|\text{θ}1} \end{array}$$

(10) $$\begin{array}{l} \theta_{22}=\theta_{21}\cdot \alpha_{\text{C}|\text{θ}2} \end{array}$$

**Figure 2:**
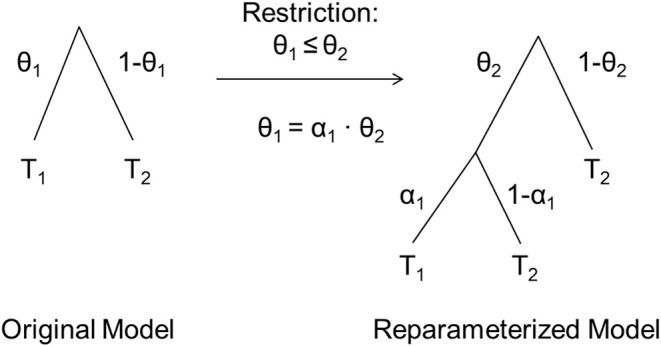
Illustrative instructions on how to reparameterize a MPT model to reflect the parametric order constraint θ_1_ ≤ θ_2_. T1 represents the sub-MPT that follows occurrences of θ_1_ in the original model, T2 the sub-MPT that follows occurrences of (1–θ_1_) in the original model. In the reparameterized model, all occurrences of θ_1_ are replaced by α_1_ · θ_2_ and (1–θ_1_) is replaced by (1–θ_2_). Sub-MPTs follow the new parameters as illustrated. Adapted from Knapp and Batchelder (2004).

Importantly, each occurrence of θ_12_ and θ_22_ in the original model is replaced with the corresponding product. The introduction of two new parameters α_C, θ1_ and α_C, θ2_ to the model of course also requires the introduction of the corresponding complementary branches (1-α_C|θ1_) and (1-α_C|θ2_). Figure 2 shows how the reparameterized model can be derived from the original model. Whereas the equations for Condition 1 correspond to those presented in Equation (1) except for an additional index “1”, for Condition 2 the reparameterized model equations become:

(11) $$\begin{array}{l} \text{p}\left({\text{f}}_{12}\right)=\theta_{11}\cdot \alpha_{\text{C}|\text{θ}1}\cdot \theta_{21}\cdot \alpha_{\text{C}|\text{θ}2}\\ \text{p}\left({\text{f}}_{22}\right)=\theta_{11}\cdot \alpha_{\text{C}|\text{θ}1}\cdot \theta_{21}\cdot \left(1-\alpha_{\text{C}|\text{θ}2}\right)+\theta_{11}\cdot \alpha_{\text{C}|\text{θ}1}\cdot \left(1-\theta_{21}\right)\\ \text{p}\left({\text{f}}_{32}\right)=\theta_{11}\cdot \left(1-\alpha_{\text{C}|\text{θ}1}\right)+\left(1-\theta_{11}\right) \end{array}$$

Obviously, the reparameterized model will always be larger (in terms of the number of branches) than the original model. Therefore, an automatized implementation of order constraints is recommended not only because it is less tedious but, more importantly, less error-prone. To our knowledge, multiTree is the only MPT modeling software that offers this automatic reparameterization of MPT models to reflect parametric order constraints. Apart from this, the reparameterized model is equivalent to the original model for the subspace of the parameter space fulfilling the implemented order constraints (cf., Meiser, 2014). Thus, the properties (e.g., identifiability; validity) of the original model, already shown by Bayen et al. (1996), also hold for the reparameterized model. Crucially note that this only pertains if the reparameterization is implemented correctly, that is if the larger parameter is in the denominator of the ratio. Indeed, in this case, and further given that the ratio parameters are not at the boundary of parameter space (i.e., 0 or 1), model fit of the reparameterized model will be identical to the original model.

Note that even though this reparameterization restricts θ_12_ and θ_22_ to vary only in [0, θ_11_] and [0, θ_21_], respectively, all parameters in the reparameterized model may vary in [0, 1], so that standard MPT procedures can be used to estimate and test the model. This is because the restricted parameters, θ_12_ and θ_22_, are not included in the reparameterized model and the new parameters, α_C|θ1_ and α_C|θ2_, reflect shrinkage factors (i.e., α_C|θ1_ = θ_12_/θ_11_ and α_C|θ2_ = θ_22_/θ_21_) that can vary in [0, 1]. If α_C|θ1_ = α_C|θ2_ = 1, then there is no condition effect on both process parameters, that is θ_12_ = θ_11_ and θ_22_ = θ_21_. In contrast if α_C|θ1_ < 1 and α_C|θ2_ < 1, then Condition 2's parameters are smaller than Condition 1's for both psychological processes of interest.

In case of a design including factors with more than two levels (e.g., three or more different psychological process parameters of interest), one would specify additional ratio parameters. For example, with three levels of the Process factor (θ_1_, θ_2_, θ_3_) and two conditions, there would be three simple main effects of Condition 1 vs. 2 (α_C|θ1_, α_C|θ2_, α_C|θ3_). Similarly, when there are more than two conditions, one would specify additional shrinkage parameters. In general, if there are *K* ≥ 2 levels of the Condition factor, then the number of shrinkage parameters required per process parameter would be *K*-1. For example, in a design with two process parameters and three conditions, one would have four shrinkage parameters in total, two for each of the two processes. The two shrinkage factors per process could, for example, represent the shrinkage in a process parameter from Conditions 1 to 2 and from Conditions 2 to 3 or, alternatively, the shrinkage from Conditions 1 to 2 and from Conditions 1 to 3. The first method would be appropriate if one expects a main effect decrease from Conditions 1 to 2 and, additionally, from Conditions 2 to 3. In contrast, if one simply expects Condition 1 to perform best with no specific ordering of Conditions 2 and 3, then one would select the second pattern of order constraints. Although the values of the second shrinkage parameter will in general differ between both methods, they are equivalent in the sense that they will yield identical interaction test results, provided that the imposed order constraints are in agreement with the observed data.

So far, we have assumed that the process effect is in the same direction across all conditions. In this case, an *ordinal* interaction would be present if this process effect significantly differs in size across conditions. In contrast, a *disordinal* interaction would be present if a factor has opposite effects across at least two levels of the other factor (also called “crossover interaction” or “double dissociation”). For example, if the Condition factor in our example from Figure 1 produced a decrease from the first to the second level in the first process parameter (i.e., θ_12_ < θ_11_) but an increase in the second parameter (i.e., θ_22_ > θ_21_) then this would establish a disordinal interaction. In turn, we will explain how to test both types of interactions.

### Testing Ordinal Two-Way Interactions in MPT Model Parameters

In ordinal interactions, the effect of one factor is in the same direction for each level of the other factor while it may vary in effect size (including zero effects). In our example, Condition 2 may always have lower process probabilities than Condition 1 irrespective of the type of process (θ_1_, θ_2_) but this difference may be more pronounced for one process than the other. To test such an interaction, one would implement the shrinkage parameters as above to reflect the effect in the expected direction at each level of the other factor. When the observed data satisfy all imposed order constraints (i.e., when an increase in the level of the Condition factor is always associated with a decrease in all relevant process parameter estimates), the model fit for the original and the reparameterized model is necessarily identical. To formally test whether there is an ordinal interaction, one then restricts the corresponding shrinkage parameters to be equal (i.e., α_C|θ1_ = α_C|θ2_). If this restriction significantly worsens model fit, that is, if the observed Δ*G*^2^(*df* ) falls in the upper α·100% of the reference distribution, using the asymptotic χ^2^(*df* ) distribution as a reference with *df* = number of equality constraints (i.e., *df* = 1 in our example), the H_0_ assuming no interaction (i.e., identical shrinkage parameters across all levels of the other factor) would be rejected in favor of the H_1_ that there is an ordinal two-way interaction.

Assuming that the interaction test turned out to be not significant, the H_0_ of no interaction is maintained. The next step would be to test the main effect of the Condition factor (restricted to be equal across processes, i.e., α_C|θ1_ = α_C|θ2_ = α_C_) for significance. The most straightforward way to do this would be to test H_0_: α_C_ = 1 using a Δ*G*^2^(*df* ) difference test with respect to the null interaction model. However, since this H_0_ lies at the boundary of the parameter space, the regularity conditions for the asymptotic χ^2^(*df* ) test are not met (Read and Cressie, 1988). This problem can be remedied using the parametric bootstrap option of multiTree (Moshagen, 2010) that generates the relevant reference distribution under H_0_ empirically using Monte-Carlo methods (Efron and Tibshirani, 1993).

If, in contrast, the interaction effect turns out to be significant, it will often be of interest to test whether the interaction is weakly ordinal with respect to a certain factor (e.g., θ_12_ < θ_11_ and θ_22_ = θ_21_, also called “simple dissociation”) or strictly ordinal (e.g., θ_12_ < θ_11_ and θ_22_ < θ_21_, also called “no dissociation”), that is, whether each of the simple main effects is significant or not. This can be done in the same way as previously described for the test of the main effect null hypothesis H_0_: α_C_ = 1. This time, however, one would test the simple main effects separately, for example, H_0_: α_C|θ1_ = 1 or H_0_: α_C|θ2_ = 1. Again, we recommend the parametric bootstrap to evaluate statistical significance.

Note that if one or both factors have three or more levels and there are no specific predictions concerning which specific combination of the two factors should differ from the other design cells, one should first conduct an omnibus test by equating all shrinkage parameters. If this effect is significant, resulting in an acceptance of the overall interaction hypothesis, one can then follow up with pairwise comparisons of the shrinkage parameters to describe this interaction in more detail. We recommend adjusting such exploratory multiple pairwise comparisons using the Bonferroni-Holm method (Holm, 1979) to prevent alpha inflation. If there are more specific a priori predictions concerning which design cells should differ from the others, then only the corresponding shrinkage parameter constraints should be tested directly to minimize the overall number of tests.

Notably, presence vs. absence of an interaction between two factors of course does not depend on the order in which these factors enter into the interaction test. Applied to our example, rather than testing the null interaction hypothesis as outlined above, that is, by equating the shrinkage parameters representing simple condition main effects (i.e., α_C|θ1_ = α_C|θ2_ = α_C_), we could also invert our reparameterization scheme and equate shrinkage parameters representing simple process main effects within conditions (i.e., α_P|C1_ = α_P|C2_ = α_P_). Both constraints are equivalent and thus lead to the same null interaction model. By implication, the goodness-of-fit statistics Δ*G*^2^(1) and test results will be identical for both types of constraint. To see the equivalence, note that α_C|θ1_ = α_C|θ2_ = α_C_ can be rewritten as:

(12) $$\begin{array}{l} \theta_{12}=\theta_{11}\cdot \alpha_{\text{C}|\text{θ}1}=\theta_{11}\cdot \alpha_{\text{C}} \end{array}$$

(13) $$\begin{array}{l} \theta_{22}=\theta_{21}\cdot \alpha_{\text{C}|\text{θ}2}=\theta_{21}\cdot \alpha_{\text{C}} \end{array}$$

Dividing (13) by (12) immediately leads to

(14) $$\begin{array}{l} \theta_{22}/\theta_{12}=\theta_{21}/\theta_{11}\Leftrightarrow \alpha_{\text{P}|\text{C}2}=\alpha_{\text{P}|\text{C}1}=\alpha_{\text{P}}. \end{array}$$

### Testing Disordinal Two-Way Interactions in MPT Model Parameters

In disordinal interactions, the direction of the effect of one factor differs across the levels of the other factor. If such an interaction holds, then a model imposing order constraints in the same direction across all levels of another factor will not fit the observed data perfectly. However, not all cases with order-constrained models resulting in *G*^2^ > 0 automatically justify acceptance of the disordinal interaction hypothesis. One possibility is that the levels of the Condition factor were just all specified in the wrong order. By re-arranging the order of factor levels one could perhaps have an order-constrained model with perfect fit to the data. This option should be checked first by inspecting raw parameter estimates for the unconstrained model. If re-arranging factor levels results in perfect fit of the order-constrained model one should proceed with this revised order model and test for an ordinal interaction as described in the previous section. Of course, such a change in the order constraints relative to the originally expected order must be reported in any publication on these data.

If inspection of the data shows that misfit of the order-constrained model cannot be avoided by re-arranging the order of factor levels, the possibility remains that a weakly ordinal interaction underlies the data but sampling error caused a disordinal interaction pattern in the observed data. Given this, the H_0_ of a weakly ordinal interaction (represented by the order-constrained model without any further constraints) cannot be evaluated using the asymptotic χ^2^(*df* ) as a reference distribution (Read and Cressie, 1988). However, one can again use the parametric bootstrap to estimate the limiting distribution of *G*^2^ under the H_0_ of an ordinal interaction. If the reparameterized model with shrinkage parameters in line with the ordinal interaction hypothesis fits the data, indicated by a bootstrapped *p*-value larger than the desired alpha-level, then the H_0_ of an ordinal interaction is maintained.

Again, in the case of three or more levels per factor one should either only test pairwise comparisons that were a priori hypothesized to differ or conduct an omnibus restricting all ratio parameters to be equal first and proceed with pairwise tests of all possible combinations only if the omnibus test is significant. As before, we recommend applying a Bonferroni-Holm correction of the alpha significance level, especially if many levels are compared.

In contrast, if the bootstrapped *p*-value is equal to or smaller than the specified alpha-level, then the H_0_ of an ordinal interaction is rejected. In other words, there are substantial effects in opposing directions, thus establishing a disordinal interaction. Again, this approach can be easily extended to factors with three or more levels by specifying as many ratio parameters as there are levels.

## Results

We now illustrate our method to test a prominent theory of cognitive aging that predicts an ordinal two-way interaction of age group and type of memory on memory performance. Specifically, the associative deficit hypothesis (Naveh-Benjamin, 2000) maintains that aging particularly impacts memory for associations such that differences between younger and older adults are most pronounced on tests of associative memory but less so on tests of simple item memory. One type of associative memory is source memory (memory for the context in which item information was first learned), such as determining whether we read information in a reliable or unreliable newspaper (Johnson et al., 1993). Thus, source memory involves memory for the association of an item to perceptual (e.g., font type) and spatio-temporal context features that together make up the source or origin of the item and should thus be more affected by aging than memory for the item itself.

In the typical source-monitoring paradigm, participants study items (e.g., words) presented in one of two different sources (e.g., in bold vs. italic text type). Subsequently, participants are tested with a list of previously studied (i.e., old) items intermixed with unstudied (i.e., new) items, all presented in a source-neutral manner (e.g., a regular text type). For each test item, participants are required to provide an old vs. new judgment. If an item is judged as old, they also have to indicate the source of the item.

**Figure 3:**
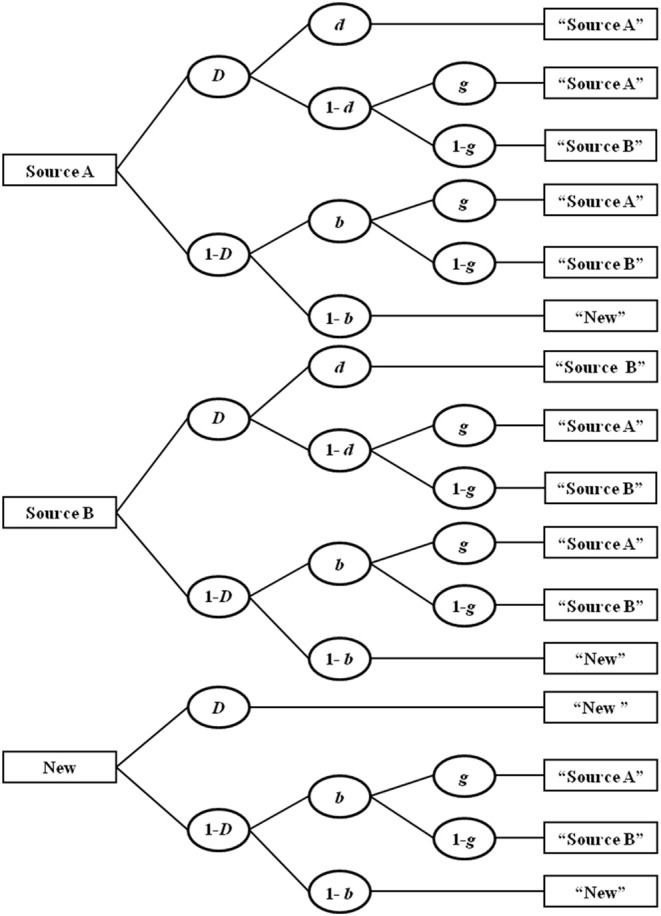
Four-parameter version of the two-high-threshold multinomial processing tree model of source monitoring. *D*, probability of recognizing a previously presented item or detecting a distractor item; *d*, probability of remembering the source an item was presented in; *b*, probability of guessing that an unrecognized item is old; *g*, probability of guessing that an item was presented in Source A. Adapted from Bayen et al. (1996, p. 202).

A well-validated MPT model commonly used for this paradigm is the two-high threshold (2HT) model of source monitoring (Bayen et al., 1996; see Bröder and Meiser, 2007, for a review of alternative MPT models). A simple variant (Submodel 4) of this model is depicted as a tree structure in Figure 3. There are three trees, one for test items that were originally studied in Source A (e.g., italic text type), another for test items originally studied in Source B (e.g., bold text type), and an additional tree for new distractor items. The model reflects the theoretical assumption that memory and guessing processes operate together in a source-monitoring task (cf., Johnson et al., 1993). That is, a correct source attribution at test for items originally studied in one of the two sources may result from actual memory for both the item (probability *D*) and the source (probability *d*), or from a series of guessing processes in the absence of memory. That is, if participants remember an item (probability *D*) but not its source (probability 1-*d*), they guess “Source A” with probability *g* and “Source B” with the complementary probability (1-*g*). In a state of uncertainty about the item (probability 1-*D*), the guessing process *b* captures the probability of guessing “old” and the complementary probability 1-*b* reflects the probability of guessing “new.” For new items, parameter *D* reflects the probability of distractor detection. In a state of uncertainty about the new item's status (with probability 1-*D*), item and, if applicable, source guesses follow as they do for previously studied but unrecognized items.

Thus, the 2HT-MPT model of source monitoring provides measures of item memory (parameter *D*) and source memory (parameter *d*) that are unconfounded by inferential guessing processes. These process parameters are thus ideal dependent variables to test the age group by memory type interaction postulated by the associative-deficit hypothesis.

Using younger and older adults' source monitoring data from Kuhlmann and Touron (2011; all-once condition) we will now illustrate how to implement our method delineated above to test the ordinal interaction predicted by the associative-deficit hypothesis. In Kuhlmann and Touron's study, 30 younger (mean age = 18.5 years) and 30 older (mean age = 67.2 years) adults studied 50 words, 25 appearing in italic text type and the remaining 25 appearing in bold text type. At test, participants judged the old-new status of 100 words (50 studied, 50 new distracters) presented in neutral text type and indicated the study text type (bold or italic) for any word judged to be old. The raw responses frequencies, aggregated across participants and items in each condition, are reported in the Appendix of Kuhlmann and Touron. We constructed a two-group version of the 2HT-MPT model of source monitoring displayed in Figure 3, yielding separate parameter estimates of the four parameters (*D, d, b*, and *g*) for the younger (subscript *YA*) and older (subscript *OA*) adults, and used multiTree (Moshagen, 2010) for parameter estimation and hypothesis tests. The data file, equation files, and multiTree file of this application example are provided as Supplementary Material.

The two-group joint standard model fit the data well, *G*^2^(4) = 6.75, *p* = 0.150. Table 1 presents parameter estimates for both age groups. Given the good fit of the model to the data, the parameter estimates may be interpreted and submitted to inferential tests. In this standard model, one can test the main effect of age group on item and source memory, respectively, via standard parameter comparisons. Restricting the item memory parameters to be equal across younger and older adults (i.e., *D*_YA_ = *D*_OA_) significantly worsens model fit, Δ*G*^2^(1) = 16.90, *p* < 0.001, showing that, as to be expected, older adults item recognition is significantly poorer than younger adults'. Likewise, restricting the source-memory parameters to be equal across younger and older adults (i.e., *d*_YA_ = *d*_OA_) significantly worsens model fit, Δ*G*^2^(1) = 8.25, *p* = 0.004. Again, older adults' source memory is significantly poorer than younger adults'. Thus, these inferential tests confirm to-be-expected simple main effects of age group on both item and source memory. However, these tests do not assess whether the age effect is more pronounced on source than on item memory as predicted by the associative deficit theory.

**Table 1:** Parameter estimates from the original and the reparameterized four-parameter version of the two-high threshold multinomial model of source monitoring for younger and older adults in Kuhlmann and Touron (2011).

**Sample**	***D***	***d***	***b***	***g***	**α_A|I_**	**α_A|S_**	***G*^2^(4)**
	Original model						6.75
Younger	0.72 (0.01)	0.38 (0.03)	0.28 (0.02)	0.50 (0.02)	–	–	
Older	0.64 (0.01)	0.25 (0.03)	0.31 (0.02)	0.54 (0.02)	–	–	
	Reparameterized model						6.75
Younger	0.72 (0.01)	0.38 (0.03)	0.28 (0.02)	0.50 (0.02)	–	–	
Older	–	–	0.31 (0.02)	0.54 (0.02)	0.89 (0.02)	0.65 (0.10)	

*D, probability of recognizing an old item or detecting a new distractor item; d, probability of remembering the source (i.e., text type) of a recognized old item; b, probability of guessing “old” if item is not recognized; g, probability of guessing “italic” if text type source is not remembered; α_A|I_, age-related decline in item recognition (relative to younger adults' item recognition); α_A|S_, age-related decline in source memory (relative to younger adults' source memory). G^2^(4) values smaller than 9.49 indicate a good model fit (p > 0.05)*.

In order to test the age group x memory type interaction, we proceeded as detailed above. Specifically, we introduced two new shrinkage parameters, α_A|I_ and α_A|S_, to reflect the effect of age group (A; in the direction OA < YA) on item (I) and source (S) memory, respectively. Therefore, older adults' item and source memory parameters are reparameterized as a proportion of younger adults' respective memory parameter as follows (see Supplementary Material for full set of model equations):

(15) $$\begin{array}{l} {\text{D}}_{\text{OA}}=\alpha_{\text{A}|\text{I}}\cdot {\text{D}}_{\text{YA}} \end{array}$$

(16) $$\begin{array}{l} {\text{d}}_{\text{OA}}=\alpha_{\text{A}|\text{S}}\cdot {\text{d}}_{\text{YA}} \end{array}$$

This reparameterized model yielded the same model fit (*G*^2^) as the original model, implying that the data satisfy the implemented same-direction order-constraints. In addition, none of the shrinkage parameter estimates converged to the boundary value 1 (or 0). We further ensured that identifiability holds for the reparameterized model, as demonstrated for the original model by Bayen et al. (1996), using multiTree's tools for identifiability check: We confirmed local identifiability by repeatedly estimating the model parameters from the Kuhlmann and Touron (2011) data 1,000 times. No deviations occurred in any of the parameter estimates (i.e., all deviations ≤ 0.00001). Similarly, the simulated identifiability analysis based on 1,000 simulations with the same number of observations per tree as in the Kuhlmann and Touron data example, yielded only minor deviations between actual and recovered model parameters beyond the second decimal (maximum deviation of −0.00198; tolerance 0.00001) in less than 0.5% of the cases (i.e., 39 out of 1,000) and an average deviation <0.00001. These rare minor deviations can be explained by numerical inaccuracies. The output files from both identifiability checks are provided in Supplementary Material.

Parameter estimates from the reparameterized model are displayed in Table 1. Note that estimates for non-reparameterized parameters (i.e., *b, g, D*_YA_, and *d*_YA_) are identical to their estimates in the original model, whereas *D*_OA_ and *d*_OA_ have been replaced by the shrinkage parameters reflecting the ratios of the original memory parameter estimates for older relative to younger adults, that is ${\hat{\alpha }}_{\text{A}|\text{I}}$ = ${\hat{D}}_{OA}$/${\hat{D}}_{YA}$ = 0.64/0.72 = 0.79, and ${\hat{\alpha }}_{\text{A}|\text{S}}$ = ${\hat{d}}_{OA}$/${\hat{d}}_{YA}$ = 0.25/0.38 = 0.65. In essence, these values represent the simple main effect estimates of age (A) on item (I) and source (S) memory, respectively. Thus, there are decreases in item and source memory with increasing adult age, as indicated by the parameter estimates ${\hat{\alpha }}_{\text{A}|\text{I}}$ < 1 and ${\hat{\alpha }}_{\text{A}|\text{S}}$ < 1. The 95% CIs for ${\hat{\alpha }}_{\text{A}|\text{I}}$, [0.85, 0.92], and for ${\hat{\alpha }}_{\text{A}|\text{S}}$, [0.45, 0.85], exclude 1, suggesting significant age-related decreases in item and source memory, in line with the earlier conducted simple main effect tests.

Most importantly, we can now directly compare the age-group effect on item vs. source memory by restricting α_A|I_ = α_A|S_ to test the age group x memory type interaction postulated predicted by the associative deficit hypothesis. Descriptively, with ${\hat{\alpha }}_{\text{A}|\text{S}}<{\hat{\alpha }}_{A|\text{I}}$, there appears to be a larger age-group difference in source (associative) memory than in item memory, as implied by this theory, and restricting both α parameters to be equal indeed significantly worsens model fit, Δ*G*^2^(1) = 4.07, *p* = 0.04, indicating that the age difference is indeed significantly larger for source than for item memory—a strictly ordinal interaction as predicted by the associative deficit theory.

## Generalization

So far, we have focused on two-way interactions. Interactions of higher orders (with three or more factors involved) imply that lower-level interactions (e.g., a two-way interaction) vary between the levels of another factor. The method of testing interactions as introduced here can be generalized to this scenario in a straightforward way. For example, we can introduce a third training factor to the experimental design illustrated in Figure 1 such that both processes were assessed in both conditions in an untrained and in a trained group. That is, there are two sets of parameter estimates per condition one for untrained and one for trained, which can be denoted by a third subscript index of “1” for untrained and “2” for trained (i.e., θ_111_ is the estimate of process θ_1_ in the untrained Condition 1 whereas θ_112_ is the estimate of process θ_1_ in the trained Condition 1). As previously, we assume a main effect of condition with lower parameter estimates in Condition 2 than 1 and a main effect of process with lower probabilities of θ_2_ than θ_1_. In addition, we now assume higher process estimates in the trained conditions for both Conditions 1 and 2. Of interest may now be to test whether this training benefit differs between Conditions 1 and 2 for one or both of the processes. That is, we are interested in testing a three-way interaction of condition, process, and training.

Given this research question, we can again make use of the reparameterization method introduced above. One way of reparameterizing the model would be to introduce the training main effect α_T_, quantifying the relative decrease in the untrained (u) compared to the trained (t) group in the first step. In a second step, the conditional (i.e., simple) main effects of condition within the two training groups, α_C|u_ and α_C|t_, respectively, would be specified. In the third and final step, one would introduce the second-order conditional main effects of process within the four condition × training combinations (α_P|C1, u_, α_P|C1, t_, α_P|C2, u_, α_P|C2, t_, respectively). Applied simultaneously, all remaining parameters are reparameterized as a function of the largest parameter, θ_112_, as follows:

(17) $$\begin{array}{l} \theta_{111}=\alpha_{\text{T}}\cdot \theta_{112}\\ \theta_{121}=\alpha_{\text{T}}\cdot \alpha_{\text{C}|\text{u}}\cdot \theta_{112}\\ \theta_{122}=\alpha_{\text{C}|\text{t}}\cdot \theta_{112}\\ \theta_{211}=\alpha_{\text{T}}\cdot \alpha_{\text{P}|\text{C}1,\text{u}}\cdot \theta_{112}\\ \theta_{212}=\alpha_{\text{T}}\cdot \alpha_{\text{P}|\text{C}1,\text{t}}\cdot \theta_{112}\\ \theta_{221}=\alpha_{\text{T}}\cdot \alpha_{\text{C}|\text{u}}\cdot \alpha_{\text{P}|\text{C}2,\text{u}}\cdot \theta_{112}\\ \theta_{222}=\alpha_{\text{T}}\cdot \alpha_{\text{C}|\text{t}}\cdot \alpha_{\text{P}|\text{C}2,\text{t}}\cdot \theta_{112} \end{array}$$

Assuming that this reparameterized model fits the data as well as the model without order constraints, several (ordinal) interaction tests can be conducted. For example, if we would impose the equality constraints α_C|u_ = α_C|t_ and α_P|C1, u_ = α_P|C1, t_ = α_P|C2, u_ = α_P|C2, t_ simultaneously (i.e., four equality constraints in total), this constraint would test the hypothesis that there are no interactions whatsoever. In other words, for each of the three factors of the design, single decrease parameters (α_T_, α_C_, and α_P_), representing the main effects of each factor, suffice to describe the relative differences between conditions, irrespective of the levels of other factors. If no specific prediction has been made before the study, this omnibus test of the H_0_ of neither two-way nor three-way interactions is the recommended first test. The three-way interaction can then be tested using the second-order conditional main effects of process within the four condition × training combinations (i.e., α_P|C1, u_, α_P|C1, t_, α_P|C2, u_, and α_P|C2, t_, respectively). For example, if α_P|C1, u_ = α_P|C1, t_ but α_P|C2, u_ ≠ α_P|C2, t_ there would be a three-way interaction such that in Condition 2 the training effect differs by process whereas in Condition 1 it does not differ by process.

Importantly, note that the order in which the parametric order constraints are implemented determines the research questions that can be tested. If one was for example interested in comparing the training benefits for each Condition x Process condition, one should have first implemented the condition and process effects and implemented the training effect as a second-order conditional main effect for each Condition × Process combination. This order also determines which two-way interactions can be tested. The reparameterization order suggested first would allow to test for the condition x training effect (i.e., α_C|u_ ≠ α_C|t_) whereas including the process effect before the training effect would allow to test for the Condition × Process interaction (i.e., α_P|C1_ ≠ α_P|C2_). In sum, the optimal order in which parameters should be reparameterized depends on the specific research hypotheses of interest.

Using this approach, a multitude of interaction test options can be derived. In fact, all types of interactions as defined in Log-Linear Models for three-way contingency tables (Read and Cressie, 1988; Agresti, 2002) can be tested in this way, the single exception being the model allowing for all possible two-way interactions (condition by process, condition by training, and training by process) but not the three-way interaction of these factors. To our knowledge, there is no way to test this specific hypothesis within the MPT framework. Despite this limitation, the approach can be used to address a large number of scientifically relevant interaction hypotheses for multi-factorial designs.

## Discussion

In this article, we explain how to test interactions on MPT model parameters. Our method relies on parametric order constraints (Knapp and Batchelder, 2004) that represent a factor's conditional main effects at each level of another factor, thereby allowing for a direct test of condition main effects against each other to test for an interaction. Crucially, parametric order constraints capture a (within- or between-subjects) factor's simple main effects as relative proportions (i.e., parameter ratios from the factor's different levels) rather than as absolute parameter differences, thus an interaction in the LLM sense is tested. We argue that the LLM concept is appropriate for MPT models because of the restriction of model parameters (i.e., probabilities) to the interval [0, 1]. Given this constraint, scaling simple main effects as absolute parameter differences would artificially imply an interaction in the presence of strong main effects, a problem that disappears when simple main effects are represented by parameter ratios. We explained in detail how both ordinal and disordinal two-way interactions on MPT model parameters can be tested. An empirical application example from cognitive aging proves the usefulness of our method to various psychological research questions. Finally, we explain how our method generalizes to higher-order interactions involving three or more factors. Our method can be easily implemented within the freely available software multiTree (Moshagen, 2010), operating on Windows, Mac and Unix. Thereby, users benefit from all other options provided by multiTree, for example the option to conduct an a priori power analysis of the interaction tests.

### Limitations

Our proposed method for testing interactions on MPT model parameters is flexible and can be easily adapted to any MPT model. Thus, it provides a powerful tool for psychological research. Nonetheless, there are a few limiting aspects that one should be aware of before implementing this method. The fact that the order of parameter restrictions determines which specific interactions can be assessed in more complex designs with three or more factors, may be perceived as a limitation of this method. However, the same applies to ANOVA models for which there are different options to follow up with simple conditional main effects analyses. As always, a good theoretical foundation of the research study should ensure that the order of implementing parameter restrictions in complex designs does not become an exploratory playground.

A more serious limitation may be the fact that one type of interaction as defined in Log-Linear Models for three-way contingency tables (Read and Cressie, 1988; Agresti, 2002) cannot be tested with this method. More specifically, it is not possible to set up a model that simultaneously allows for all possible two-way interactions (Factor 1 × Factor 2, Factor 1 × Factor 3, Factor 2 × Factor 3) but omits the three-way interaction of these factors (Factor 1 × Factor 2 × Factor 3). Nonetheless, all other types of interactions can be tested and as there are ways to specifically test each two-way interaction, we think this does not pose a real limitation to the applicability of MPT interaction tests for nearly all psychological research questions.

Finally, we have focused on MPT model applications based on data (i.e., observed frequencies) aggregated across items and participants within each experimental condition, as is traditionally done in MPT research (Batchelder and Riefer, 1999; Chechile, 2009; Erdfelder et al., 2009). A potentially problematic aspect of this “complete pooling” approach is that observations are treated as independently and identically distributed across the response categories. More recent approaches, mostly based on Bayesian methods and partial pooling, have been developed to estimate MPT parameters in the presence of heterogeneity among observations (Klauer, 2006, 2010; Stahl and Klauer, 2007; Smith and Batchelder, 2008, 2010; Matzke et al., 2015; Heck et al., 2018). Notably, as our approach to test interactions is based on reparameterized standard MPT models, it can also be used in combination with all of these hierarchical MPT approaches as long as no between-subjects factor is involved. When Bayesian hierarchical MPT models are used, however, carefully note recommendations regarding prior distributions for order-constrained parameters as standard settings may not be appropriate (Heck et al., 2015).

We recommend that users test whether heterogeneity is present in their frequency data (see Smith and Batchelder, 2008, for such tests). If significant heterogeneity is present, parameter estimates based on complete pooling should be compared with partial-pooling approaches to assess potential risks of aggregation bias in their data. Note that particularly in experimental MPT research, complete pooling of data may have advantages even if some moderate degree of heterogeneity is present in the data (e.g., Chechile, 2009).

## Conclusion

In summary, MPT models are valuable measurement tools for psychological research (cf., Erdfelder et al., 2009) but its previous applications have been primarily restricted to analyses of main effects only. With the method outlined in this article, various types of interactions can be tested on MPT model parameters, rendering this tool even more powerful for future psychological research.

## Data Availability Statement

The aggregated raw response frequency data are provided in the Appendix of Kuhlmann and Touron (2011). We additionally provide this data file as well as all our analysis files in the Supplementary Material. The individual response frequencies can be obtained from BK, kuhlmann@psychologie.uni-mannheim.de.

## Author Contributions

BK drafted the manuscript, provided the empirical data, and conducted the statistical analyses. All three authors contributed equally to the methodological aspects of this paper and improved and revised the manuscript conjointly.

### Conflict of Interest

The authors declare that the research was conducted in the absence of any commercial or financial relationships that could be construed as a potential conflict of interest.

## Footnotes

^1^For a more elaborated MPT model of the processes involved in the Remember-Know recognition task see Erdfelder et al. (2007).

^2^Because invariant parameter ratios correspond to invariant differences between the logarithms of the parameters, a technically more accurate term would be “interaction on a logarithmic scale” (cf. Klauer et al., 2000, p. 861). In fact, Klauer and collaborators already employed the LLM concept of no interaction in the context of their belief bias MPT model (Klauer et al., 2000), albeit without discussing its implementation in MPT models and its implications for MPT modeling in general.

## Supplementary Material

Supplementary Material Data and analyses files.
